# *Polygonum cuspidatum* Extract Induces Anoikis in Hepatocarcinoma Cells Associated with Generation of Reactive Oxygen Species and Downregulation of Focal Adhesion Kinase

**DOI:** 10.1155/2012/607675

**Published:** 2012-09-13

**Authors:** Bing Hu, Hong-Mei An, Ke-Ping Shen, Hai-Yan Song, Shan Deng

**Affiliations:** ^1^Department of Oncology and Institute of Traditional Chinese Medicine in Oncology, Longhua Hospital, Shanghai University of Traditional Chinese Medicine, Shanghai 200032, China; ^2^Department of Science and Technology, Longhua Hospital, Shanghai University of Traditional Chinese Medicine, Shanghai 200032, China; ^3^Institute of Digestive Disease, Longhua Hospital, Shanghai University of Traditional Chinese Medicine, Shanghai 200032, China

## Abstract

Anoikis has been recognized as a potential target for anticancer therapy. *Polygonum cuspidatum* (Huzhang) is a frequently used Chinese herb in hepatocarcinoma. In present study, we evaluated the effects of *Polygonum cuspidatum* extract (PCE) in hepatocarcinoma cells in suspension. The results showed that PCE inhibited the proliferation of hepatocarcinoma cells in suspension in a dose- and time-dependent manner. PCE also inhibited anchorage-independent growth of hepatocarcinoma cells in soft agar. PCE induced anoikis in human hepatocarcinoma Bel-7402 cells accompanied by caspase-3 and caspase-9 activation and poly(ADP-ribose) polymerase cleavage, which was completely abrogated by a pan caspase inhibitor, Z-VAD-FMK. In addition, PCE treatment induced intracellular reactive oxygen species (ROS) production in Bel-7402 cells. NAC, an ROS scavenger, partially attenuated PCE-induced anoikis and activation of caspase-3 and caspase-9. Furthermore, PCE inhibited expression of focal adhesion kinase (FAK) in Bel-7402 cells. Overexpression of FAK partially abrogated PCE-induced anoikis. These data suggest that PCE may inhibit suspension growth and induce caspase-mediated anoikis in hepatocarcinoma cells and may relate to ROS generation and FAK downregulation. The present study provides new insight into the application of Chinese herb for hepatocarcinoma treatment.

## 1. Introduction

Epithelial cells require attachment to the extracellular matrix to provide survival signal. Detachment from the extracellular matrix causes apoptosis, a process known as anoikis, or detachment-induced apoptosis. Anoikis is a Greek word that means homelessness, as apoptotic process; anoikis was first described by Frisch and Francis in 1994 [1]. Physiologically, anoikis played a critical role in organismal development and homeostasis [1]. Pathophysiologically, resistance to anoikis is acquired in epithelial cancer cells due to gene expression or activity abnormality, which allows cancer cells to survive in an anchorage-independent manner when deprived of extracellular matrix attachment during dissemination in blood or lymph, and associated with metastatic spread of cancer cells [2]. Anoikis has been suggested as a potential target for anticancer therapy [3, 4].

Hepatocarcinoma is one of the most frequent malignancies and remains the third leading cause of cancer death worldwide [5–7]. Huzhang (*Polygonum cuspidatum*) is a well-tolerated Chinese herb used for treating liver diseases with damp-heat and blood-stasis syndrome and has been regarded as an anticancer herb in modern traditional Chinese medicinal practice and frequently used in hepatocarcinoma. *Polygonum cuspidatum* has displayed anticancer effects in oral cancer and lung cancer cells [8, 9]. The active chemical ingredients of *Polygonum cuspidatum* include resveratrol, emodin, polydatin, and physcion [10, 11]. Resveratrol and emodin have shown anticancer potential in various cancer cells, including hepatocarcinoma cells [12, 13]. Emodin may induce reactive oxygen species (ROS) and sensitize gastric carcinoma cells to anoikis [14]. We have shown that a *Polygonum cuspidatum* containing Chinese herbal formula may inhibit suspension growth and induce anoikis in hepatocarcinoma cells [15]. However, the effect of *Polygonum cuspidatum* against hepatocarcinoma cells in suspension remains unknown.

In present study, we evaluated anticancer potential of *Polygonum cuspidatum* against hepatocarcinoma cells in suspension growth. The results showed that *Polygonum cuspidatum* extract (PCE) inhibited suspension growth, activated caspases, and induced anoikis in hepatocarcinoma cells, and may relate to ROS generation and downregulation of focal adhesion kinase (FAK).

## 2. Materials and Methods

### 2.1. Chemicals and Reagents

CytoSelect 24-Well Anoikis Assay was provided by Cell Biolabs (San Diego, CA). Poly-HEMA was from Sigma-Aldrich (St. Louis, MO). Colorimetric CaspACE Assay System was the product of Promega (Madison, WI). Z-VAD-FMK, Caspase-8, and Caspase-9 Colorimetric Assay kits were from R&D Systems (Minneapolis, MN). Antibodies against poly(ADP-ribose) polymerase (PARP), FAK, Phospho-FAK, and GAPDH were obtained from Cell Signaling Technology (Danvers, MA). Cell Counting Kit-8 was purchased from Dojindo (Kumamoto, Japan). 2′,7′-dichlorofluorescin diacetate (DCFH-DA) and N-acetylcysteine (NAC) were provided by Beyotime Institute of Biotechnology (Jiangsu, China). Recombinant eukaryotic expression plasmid encoding full length of human FAK (re-FAK) and empty vector was obtained from Genechem (Shanghai, China). Lipofectamine 2000 was from Invitrogen (Carlsbad, CA). Polydatin, Resveratrol Emodin, Physcion was purchased from Shanghai R&D Centre for standardization of Chinese Medicines (Shanghai, China), and the purity was higher than 98.0%.

### 2.2. Herbal Preparation

PCE was prepared as a lyophilized-dry powder of hot water extracts as described previously [15]. Authentic herb materials were provided by Longhua Hospital herb store. *Polygonum cuspidatum* (100 g) was soaked for 1 h and decocted twice with 8-fold volume of distilled water for 2 h. The decoction was filtered and centrifuged twice at 12000 rpm for 30 min to remove insoluble ingredients. The supernatants were mixed with an equal volume of ethanol and kept at 4°C overnight and centrifuged at 12000 rpm for 30 min to remove insoluble ingredients. The resultant supernatants were lyophilized, weighed, dissolved in RPMI1640 medium, and adjusted to a concentration of 400 mg/mL and were sequentially passed through 0.45 *μ*m and 0.22 *μ*m filters sterilization. The average yield of PCE obtained was 7.32%. Active compounds in PCE were detected by High Performance Liquid Chromatography. Chromatographic separations were carried out on a Merck C18 Hibar column (4.6 mm × 250 mm, 5 *μ*m) as described [10]. The presence and proportion of the main constituents of PCE were identified as resveratrol (2.59%), emodin (7.84%), polydatin (5.10%), and physcion (5.50%).

### 2.3. Cell Culture

Human hepatocarcinoma cell line Bel-7402 and murine hepatocarcinoma cell line Hepa 1–6 were obtained from Cell Bank of Type Culture Collection of Chinese Academy of Sciences. Bel-7402 and Hepa 1–6 cells were grown in RPMI1640 medium with 10% FBS and 1% Pen-Strep and maintained at a 37°C in a humidified incubator with a 5% CO_2_ atmosphere.

### 2.4. Anchorage-Independent Growth Assay

Cells in logarithmic growth phase were seeded into Poly-HEMA coated (10 mg/mL) 96-well plate (5 × 10^3^ cells/well). After 24 h cells were exposed to various doses of PCE or equal volume of RPMI1640. At the end of treatment, the floated cells were collected, and cell viability was evaluated by using the Cell Counting Kit-8 assay according to the manufacturer's instructions. The cell survival rate was calculated as follows: cell survival rate (%) = (experimental OD value/control OD value) × 100%.

### 2.5. Soft Agar Colony Formation Assays

For the soft agar colony formation assays, 2 × 10^4^ log-phase hepatocarcinoma cells were seeded and grown on a plate containing 1% base agar and 0.6% top agar and exposed to different concentrations of PCE or equal volume of RPMI1640 twice a week for 2 weeks and incubated at 37°C in a humidified incubator with a 5% CO_2_ atmosphere. Colonies were stained with crystal violet and counted under a dissecting microscope. The inhibition of colony formation was calculated as follows: inhibition of colony formation (%) = (1 − experimental colony number/control colony number) × 100%.

### 2.6. Anoikis Assay

Anoikis was detected by CytoSelect 24-Well Anoikis Assay according to the manufacturer's instructions. Briefly, log-phase hepatocarcinoma cells (3 × 10^4^ cells/well) were inoculated in Poly-HEMA coated 24-well plate. On the second day, the cells were exposed to different dose of PCE or equal volume of RPMI1640 for 24 h. The floated PCE-treated or control cells were collected and stained with ethidium homodimer (EthD-1) at 37°C for 1 h. The presence of red EthD-1 fluorescence was monitored under a fluorescence microscope and quantitated with a fluorescence microplate reader at excitation wavelength of 525 nm and emission wavelength of 590 nm.

### 2.7. Flow Cytometric Assays

Cells were treated as indicated, collected, and stained with Annexin V-FITC and PI as recommended by the manufacturer (BD Biosciences). Apoptotic cells were detected in a FACScalibur flow cytometer (Becton Dickinson).

### 2.8. Caspase Activation Assay

After treatment for the indicated time with different concentration of PCE, caspase-3, 8, 9 activities were measured by the cleavage of the specific chromogenic substrate according to manufacturer's instructions. For caspases inhibition, cells pretreated with Z-VAD-FMK (50 *μ*mol/L, 2 h) were incubated with PCE for another 24 h.

### 2.9. Western Blot

Western blots were performed as described previously [15, 16]. Briefly, collected cells were lysed and subjected to 8–10% SDS-PAGE gel and transferred into a nitrocellulose membrane (Amersham). The transferred membrane was blocked with 5% nonfat milk, washed, and probed with antibodies against PARP (1 : 1000), FAK (1 : 1000), Phospho-FAK (1 : 1000), or GAPDH (1 : 2000). Blots were then washed and incubated with IRDye 700-conjugated (1 : 3000) or IRDye 800-conjugated (1 : 5000) secondary antibodies (Rockland Immunochemicals) and visualized in Odyssey Infrared Imaging System (LI-COR Biosciences).

### 2.10. Measurement of Intracellular ROS Levels

Intracellular ROS production was detected by DCFH-DA staining. DCFH-DA is cleaved intracellularly by nonspecific esterases to form DCFH, which is further oxidized by ROS to form the fluorescent compound DCF [17]. Log-phase hepatocarcinoma cells (2 × 10^5^ cells/well) were seeded in Poly-HEMA coated 6-well plate. On the second day, the cells were exposed to different dose of PCE or equal volume of RPMI1640 for 24 h and stained with DCFH-DA at 37°C for 20 minutes in the dark. The presence of DCF fluorescence was quantitated with a fluorescence microplate reader at excitation wavelength of 488 nm and emission wavelength of 525 nm. For ROS inhibition, cells were pretreated with NAC (50 mmol/L for 2 h), followed by desired PCE treatment.

### 2.11. Plasmid Transfection

For plasmid transfection, Bel-7402 cells were cultured on 6-well plate to 90–95% confluence, and 4.0 *μ*g recombinant human FAK eukaryotic expression plasmid or control empty vector was introduced into the cells using Lipofectamine 2000 according to the manufacturer's recommendations. After 24 h of transfection, cells were subjected to anoikis assay, ROS detection, and western blot.

### 2.12. Statistical Analysis

Results are expressed as means ± standard deviation of at least two independent experiments, each conducted in triplicate. Differences between control and PCE treatment were analyzed by 1-way ANOVA. Differences were considered significant at *P* < 0.05.

## 3. Results

### 3.1. PCE Inhibited Proliferation of Hepatocarcinoma Cells in Suspension

**Figure 1:**
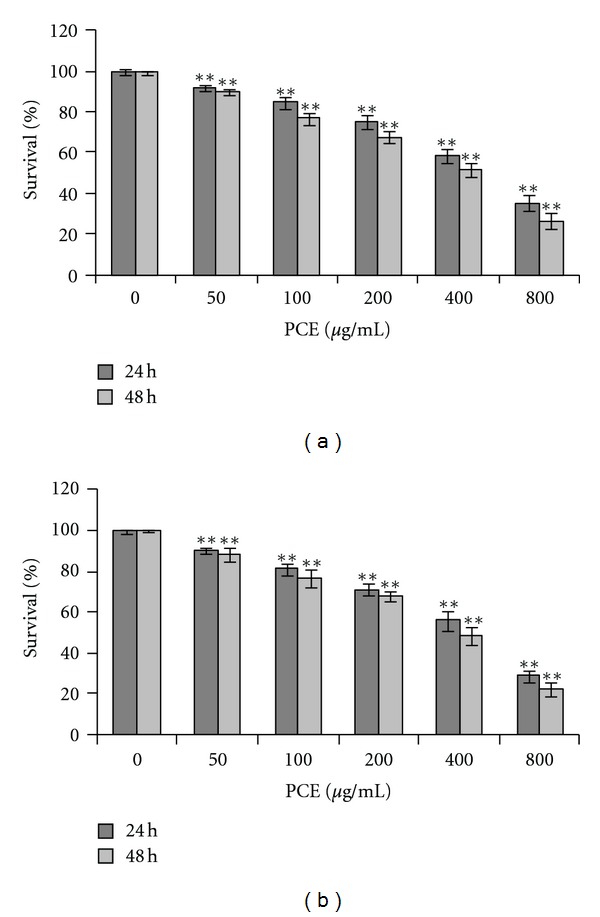
PCE inhibited proliferation of hepatocarcinoma cells in suspension. Human hepatocarcinoma Bel-7402 cells (a) and murine hepatocarcinoma Hepa 1–6 cells (b) were treated with different concentrations of PCE; cell viability was evaluated by CCK-8 assay. Data shown are representative of three independent experiments. **P < 0.01, versus control group.

The effects of PCE on cell growth of hepatocarcinoma cells in suspension were detected by using Poly-HEMA coated plate in which cell grew in an anchorage-independent manner [1, 18]. Human hepatocarcinoma cell line Bel-7402 and murine hepatocarcinoma cell line Hepa 1–6 from different species were used as model cells. The results showed that PCE, at a concentration of 50–800 *μ*g/mL, significantly inhibited proliferation of Bel-7402 cells and Hepa 1–6 cells in suspension in a dose- and time-dependent manner (Figure 1) (*P* < 0.05).

### 3.2. PCE Inhibited Colony Formation of Hepatocarcinoma Cells in Soft Agar

**Figure 2:**
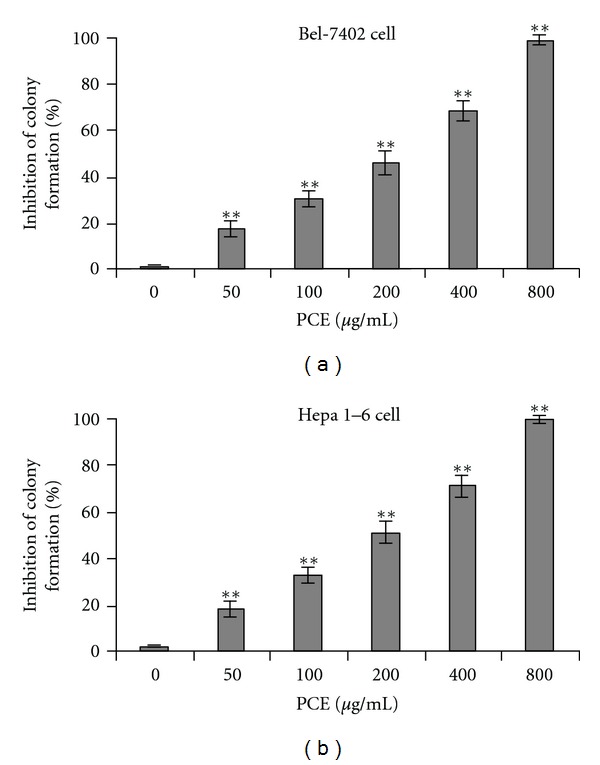
PCE inhibited colony formation of hepatocarcinoma cells in soft agar. Bel-7402 cells (a) and Hepa 1–6 cells (b) were treated with different dose of PCE twice a week for 2 weeks in the soft-agar colony formation assay. The colonies were stained and counted. Data shown are representative of three independent experiments (each conducted in triplicate). **P < 0.01, versus control group.

Long-term effects of PCE on anchorage-independent cell growth were further investigated in soft agar colony formation assays. As shown in Figure 2, colony formation of Bel-7402 cells and Heap 1–6 cells was significantly inhibited by PCE treatment in a dose-dependent manner (*P* < 0.01). At higher dose (800 *μ*g/mL) of PCE treatment, the inhibition of colony formation in Bel-7402 cells and Heap 1–6 cells was 98.60% and 99.07%, respectively.

### 3.3. PCE Induced Anoikis in Hepatocarcinoma Cells

**Figure 3:**
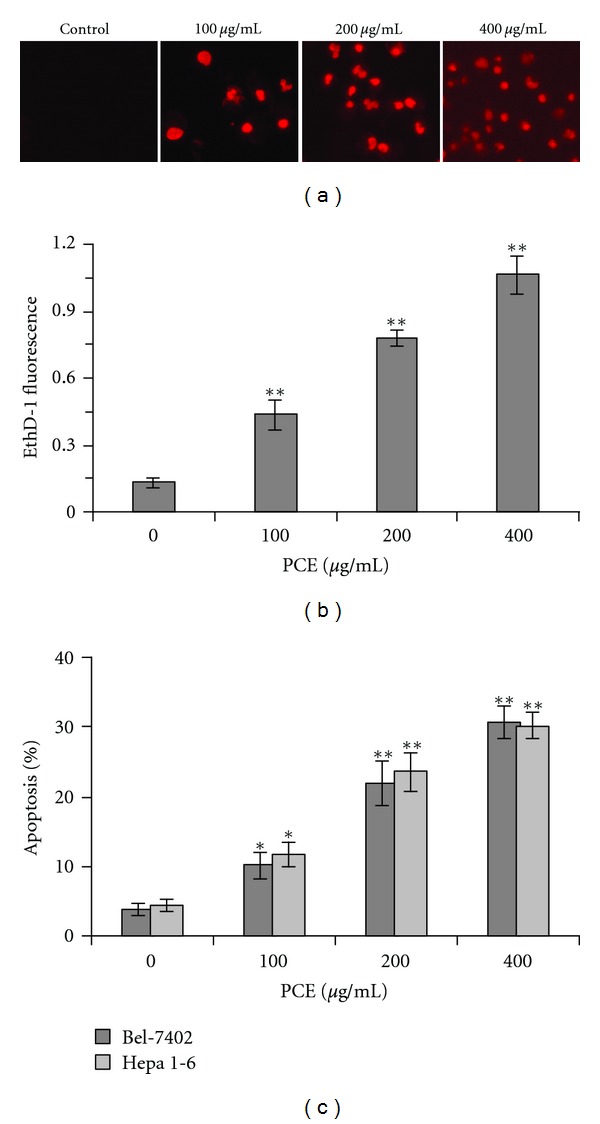
PCE induced anoikis in hepatocarcinoma cells. (a) Bel-7402 cells were treated with different dose of PCE for 24 h in Poly-HEMA coated plates, stained with EthD-1, and observed under fluorescence microscope (×100). (b) The fluorescence of EthD-1 absorbed by Bel-7402 cells was detected with fluorescence microplate reader and expressed as mean ± SD. (c) PCE-treated Bel-7402 cells and Hepa 1–6 cells were stained with Annexin V-FITC/PI and detected in FACScalibur flow cytometer. Data illustrated are from three separate experiments. *P < 0.05, versus control group. **P < 0.01, versus control group.

EthD-1, a red fluorescent dye, was used to detect cell death in suspension culture. As shown in Figures 3(a) and 3(b), after PCE treatment, EthD-1 was absorbed by Bel-7402 cells yielding a red-fluorescent nuclear staining. In addition, the photodensity of the red fluorescence between PCE, in different dosage and control groups, showed a significant difference (*P* < 0.01). Flow cytometry analysis was used to further discriminate necrosis and apoptosis. As shown in Figure 3(c), PCE significantly induced apoptosis in Bel-7402 cells and Hepa 1–6 cells in suspension culture (*P* < 0.05). These results suggested PCE may induce anoikis in hepatocarcinoma cells.

### 3.4. PCE Activated Caspases in Bel-7402 Cells

**Figure 4:**
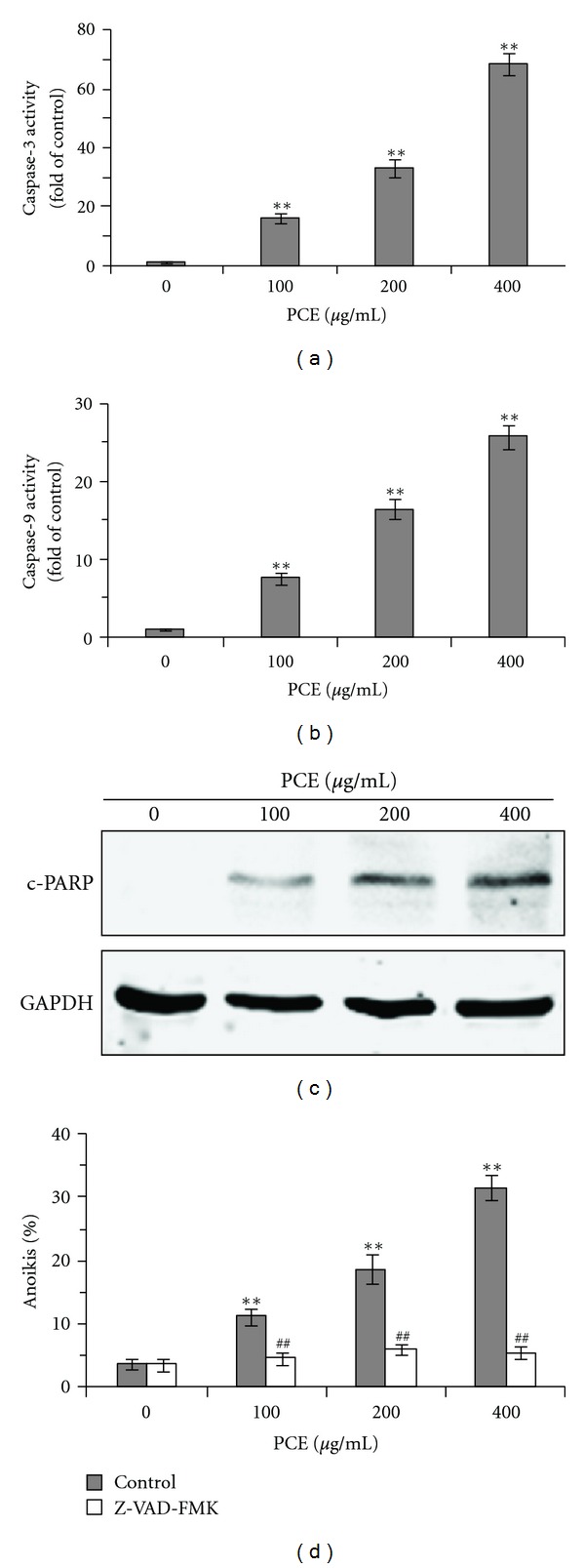
PCE-activated caspases in Bel-7402 cells. After 24 h PCE (100–400 *μ*g/mL) treatment, caspase-3 (a) and caspase-9 (b) activity in suspension-cultured Bel-7402 cells were detected as described in Materials and Methods Section. Caspases activities were expressed as fold activation over control. (c) Cleaved PARP (c-PARP) was detected by Western blotting with specific antibody. GAPDH was used as a loading control. (d) Suspension-cultured Bel-7402 cells were pretreated with Z-VAD-FMK (50 *μ*mol/L) for 2 h before treatment with PCE for 24 h, stained with Annexin V-FITC/PI, and analyzed by flow cytometry. Data presented are from three separate experiments. **P < 0.01, versus control group; ^##^ *P* < 0.01, Z-VAD-FMK groups versus corresponding dose of PCE-treated (control) group.

Activation of caspases (cysteine aspartate-specific proteinase) has been recognized as hallmarks of apoptosis. Anoikis, a type of apoptosis in suspension, is also executed by caspases cascade [2, 19]. To determine whether caspases attributed to PCE-induced apoptosis in Bel-7402 cells during suspension growth, activities of caspase-3, 8, and 9 were detected. As shown in Figures 4(a) and 4(b), PCE activated caspase-3 and 9 in a dose-dependent manner and was compared with controls (*P* < 0.01). However, activity of caspase-8 was not significantly changed after PCE treatment (data not shown). In addition, PARP, one of the earliest substrates of caspase-3 during apoptosis [20], was also cleavaged after PCE treatment (Figure 4(c)). Furthermore, Z-VAD-FMK, a pan caspases inhibitor, significantly inhibited PCE-induced anoikis in Bel-7402 cells (*P* < 0.01) (Figure 4(d)), which indicated that the anoikis-inducing effect of PCE was dependent on caspases activation.

### 3.5. PCE Upregulated ROS Level in Bel-7402 Cells

**Figure 5:**
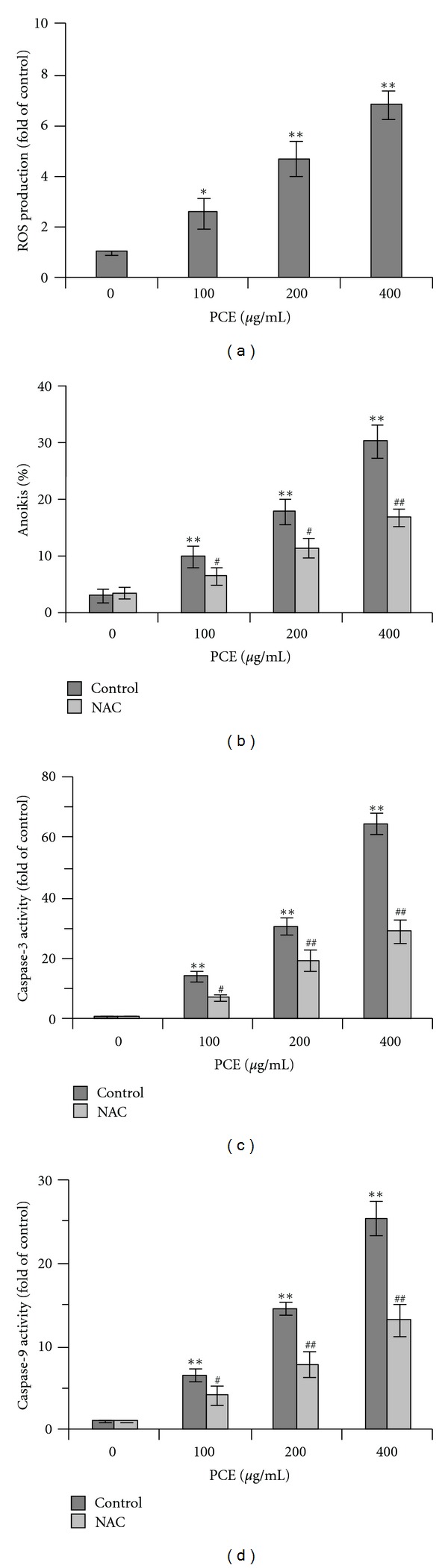
PCE upregulated ROS level in Bel-7402 cells. (a) After 24 h PCE (100–400 *μ*g/mL) treatment, intracellular ROS production in suspension-cultured Bel-7402 cells was detected as described in Materials and Methods section. ROS level was expressed as fold activation over control. For ROS inhibition, suspension-cultured Bel-7402 cells were pretreated with NAC (50 mmol/L for 2 h), followed by PCE (100–400 *μ*g/mL) treatment for 24 h, and subjected to apoptosis (b), caspase-3 (c), and caspase-9 (d) activity assay. Caspases activities were expressed as fold activation over control. Data shown are representative of three independent experiments. *P < 0.05, versus control group; **P < 0.01, versus control group; ^#^ *P* < 0.05, NAC groups versus corresponding dose of PCE-treated (control) group; ^##^ *P* < 0.01, NAC groups versus corresponding dose of PCE-treated (control) group.

It has been reported ROS is an important mediator of anoikis and related to caspases activation [21]. Natural products, such as emodin and curcumin, may sensitize cancer cell to anoikis through ROS generation [14, 22]. So we further tested the effects of PCE on ROS production. Using ROS sensitive fluorescent probe, we found PCE treatment induced intracellular ROS production in Bel-7402 cells in a dose-dependent manner and was compared with controls (*P* < 0.05) (Figure 5(a)). NAC, an ROS scavenger, partially but significantly abrogated PCE-induced anoikis in Bel-7402 cells (*P* < 0.05) (Figure 5(b)). Furthermore, PCE-induced activation of caspase-3 and caspase-9 was significantly reduced by NAC pretreatment (*P* < 0.05) (Figures 5(c) and 5(d)). These results suggested PCE inducing caspases activation and anoikis is associated with ROS generation.

### 3.6. PCE Downregulated FAK in Bel-7402 Cells

**Figure 6:**
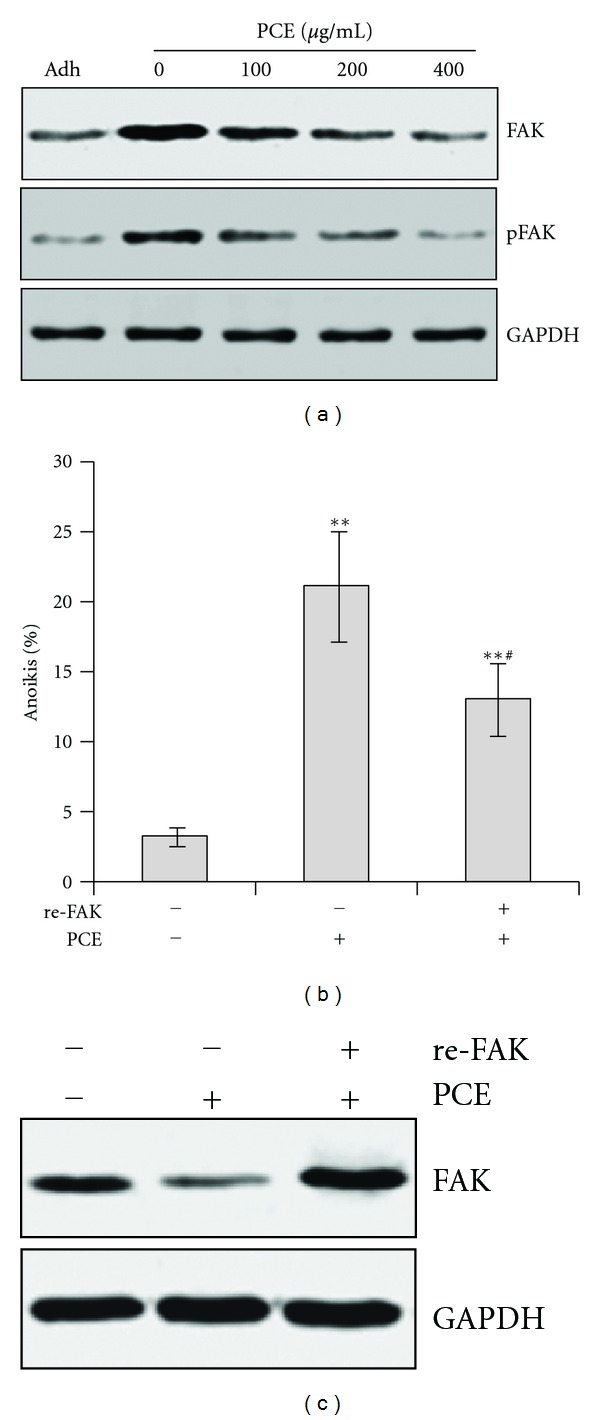
PCE inhibited FAK expression in Bel-7402 cells. (a) Adherent growth Bel-7402 cells (Adh) and PCE-treated suspension-cultured Bel-7402 cells were collected and subjected to western blots using antibody against FAK and Phospho-FAK (pFAK). (b) Bel-7402 cells were transfected with recombinant human FAK and empty vector and subjected to suspension-culture, PCE (200 *μ*g/mL) treatment and anoikis assay. At the same time, FAK expression was determined by western blot (c). GAPDH was used as a loading control. **P < 0.01, versus control group; ^#^ *P* < 0.05, re-FAK groups versus PCE-treated empty vector group.

In cancer cell, detachment from the extracellular matrix may activate FAK and resistance to anoikis [23]. We also evaluated the effects of PCE on FAK expression. As shown in Figure 6(a), low level of expression and phosphorylation of FAK was detected in Bel-7402 cells. Upon detachment from adherence, expression and phosphorylation of FAK were upregulated. After PCE treatment, expression and phosphorylation of FAK were significantly decreased in dose-dependent manner. To examine whether downregulation of FAK contributes to PCE-induced anoikis, a recombinant eukaryotic expression plasmid encoding full length of human FAK (re-FAK) was transfected to Bel-7402 cells. As shown in Figure 6(b), FAK was overexpressed in re-FAK transfected Bel-7402 cells. FAK over-expression partially but significantly abrogated PCE-induced anoikis and was compared with controls (*P* < 0.05). These observations suggested FAK downregulation contributed to PCE-induced anoikis.

### 3.7. The Relation between PCE-Induced ROS and FAK Downregulation

**Figure 7:**
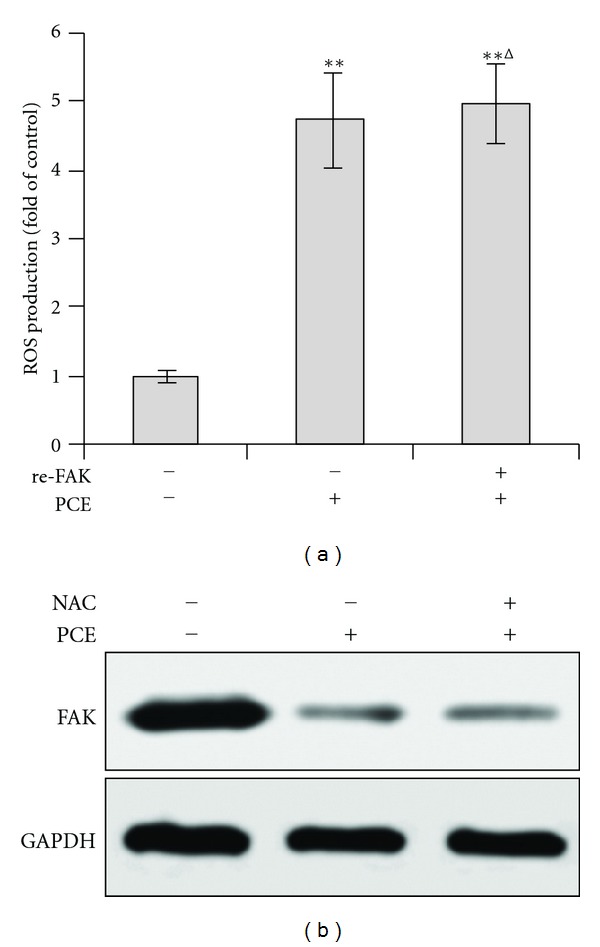
The relation between PCE-induced ROS and FAK downregulation. (a) Bel-7402 cells were transfected with recombinant human FAK and empty vector and subjected to suspension culture, PCE (200 *μ*g/mL) treatment, and ROS assay. (b) NAC pretreated or untreated Bel-7402 cells were treated with PCE (200 *μ*g/mL) and subjected to western blot. **P < 0.01, versus control group; ^∆^ *P* > 0.05, re-FAK group versus PCE-treated empty vector group.

To elucidate the relation between PCE-induced ROS and FAK downregulation, FAK overexpressed Bel-7402 cells were treated with PCE and subjected to ROS detection. The result showed PCE-elicited ROS production was not changed upon FAK over-expression (Figure 7(a)). Furthermore, abrogation of ROS production by NAC pretreatment has no effect on PCE-induced FAK downregulation (Figure 7(b)). These observations suggested ROS production and FAK downregulation were independent events in PCE-induced anoikis in Bel-7402 cells. 

## 4. Discussion

Poly-HEMA, a nontoxic polymer of 2-hydroxyethyl methacrylate, was extensively utilized to deprive anchorage *in vitro* because of its ability to reduce tissue culture plastic adhesivity [1, 18, 24]. Our present results showed that PCE can inhibit Bel-7402 cells and Heap 1–6 cells proliferation in the Poly-HEMA mimicked detachment from the extracellular matrix. Soft agar colony formation assay further demonstrated that PCE significantly inhibited colony forming capacity of Bel-7402 cells and Heap 1–6 cells in soft agar without adherence. These observations suggested PCE could inhibit Bel-7402 cells and Heap 1–6 cells growth in suspension.

EthD-1 is a high-affinity nucleic acid fluorescent dye, which can only penetrate cells once the membrane is damaged (a hallmark of dead cells) and produces bright red fluorescence upon binding to nucleic acids, and could be used to detect anoikis. In present study, EthD-1 staining indicated that partial Bel-7402 cells absorbed EthD-1 and emitted red fluorescence after PCE treatment. In addition, PI/Annexin V staining and flow cytometric assays further confirmed PCE may induce apoptosis in suspension-cultured hepatocarcinoma cells. These observations suggest that PCE may induce anoikis in Bel-7402 cells.

Similar to classic apoptosis, anoikis is also executed by intracellular caspases that are activated during the onset of apoptosis by extrinsic and intrinsic pathways [2, 4, 19]. The extrinsic pathway involves oligomerization of cell-surface death receptors by their ligands, resulting in recruitment and activation of caspase-8 followed by activation of executioner caspases-3. The intrinsic pathway involves the signals to mitochondria, which lead to the release of cytochrome c, Apaf-1, forming an apoptosome that activates the initiating protease caspase-9 which in turn activates executioner caspases-3, causing the cell to undergo apoptosis. The present study demonstrated that PCE activated caspase-3 and caspase-9 in Bel-7402 cells. In addition, PARP, one of the earliest substrates of caspase-3 during apoptosis [20], was also cleavaged after PCE treatment. Furthermore, blocking caspases activity completely abrogated PCE-induced anoikis. These results suggest that the anoikis-inducing effects of PCE were related to intrinsic apoptotic pathway.

Many natural products, such as casticin, curcumin, wogonin, Tanshinone IIA, and berberine, cause apoptosis through the mediation of ROS [25–29]. Emodin and curcumin may sensitize cancer cell to anoikis through ROS generation [14, 22]. The present results indicate that the anoikis induced by PCE in hepatocarcinoma cells is triggered by ROS-dependent activation of intrinsic apoptotic pathway. This conclusion is based on the following observations: (1) PCE treatment caused a dose-dependent ROS production in Bel-7402 cells; (2) PCE-induced anoikis was significantly attenuated by ROS scavenger NAC; (3) PCE-induced activation of caspase-3 and caspase-9 was significantly reduced by NAC pretreatment. Since NAC pretreatment only partially abrogated PCE-induced anoikis in Bel-7402 cells, there were other mechanism suggested to participate in PCE-induced anoikis.

Epithelial cells require attachment to the extracellular matrix to provide survival signal. In cancer cell, detachment from the extracellular matrix may activate FAK and resistance to anoikis [23]. Downregulation of FAK may promote cancer cell anoikis [30, 31]. The present results demonstrated that PCE-induced anoikis in Bel-7402 cells in coincidence with FAK downregulation. In addition, FAK over-expression partially abrogated PCE induced anoikis. These observations suggested PCE-induced anoikis may be related to FAK. Nevertheless, further studies are needed to determine the upstream regulator and downstream effectors of FAK.

## 5. Conclusion

In conclusion, our findings demonstrated PCE may inhibit suspension growth, activate caspases, and induce anoikis in hepatocarcinoma cells and may relate to ROS generation and FAK downregulation. Since anoikis is associated with cancer metastasis and cell survival in blood or lymphatic circulation, the effects of PCE against hepatocarcinoma metastasis and hepatocarcinoma cells in blood or lymphatic circulation are worthy of further study. The present study provides new insight into the application of Chinese herb for hepatocarcinoma treatment that is worthy of further study.
